# China’s natural carrying capacity: its change and progress of decision-oriented study

**DOI:** 10.1093/nsr/nwag205

**Published:** 2026-04-02

**Authors:** Jie Fan, Jianxiong Wu, Xiang Gao, Baoyin Liu, Kan Zhou, Dong Chen, Yafei Wang, Rui Guo, Hanchu Liu, Daojing Zhou

**Affiliations:** Institute of Geographic Sciences and Natural Resources Research, Chinese Academy of Sciences, Beijing 100101, China; Institutes of Science and Development, Chinese Academy of Sciences, Beijing 100101, China; College of Resources and Environment, University of Chinese Academy of Sciences, Beijing 100101, China; Institute of Geographic Sciences and Natural Resources Research, Chinese Academy of Sciences, Beijing 100101, China; College of Resources and Environment, University of Chinese Academy of Sciences, Beijing 100101, China; Institute of Geographic Sciences and Natural Resources Research, Chinese Academy of Sciences, Beijing 100101, China; College of Resources and Environment, University of Chinese Academy of Sciences, Beijing 100101, China; Institutes of Science and Development, Chinese Academy of Sciences, Beijing 100101, China; Institute of Geographic Sciences and Natural Resources Research, Chinese Academy of Sciences, Beijing 100101, China; College of Resources and Environment, University of Chinese Academy of Sciences, Beijing 100101, China; Institute of Geographic Sciences and Natural Resources Research, Chinese Academy of Sciences, Beijing 100101, China; College of Resources and Environment, University of Chinese Academy of Sciences, Beijing 100101, China; Institute of Geographic Sciences and Natural Resources Research, Chinese Academy of Sciences, Beijing 100101, China; College of Resources and Environment, University of Chinese Academy of Sciences, Beijing 100101, China; Institutes of Science and Development, Chinese Academy of Sciences, Beijing 100101, China; Institutes of Science and Development, Chinese Academy of Sciences, Beijing 100101, China; Institutes of Science and Development, Chinese Academy of Sciences, Beijing 100101, China

**Keywords:** natural carrying capacity, carrying system, carried entities, sustainability, decision-oriented

## Abstract

International research on planetary boundaries and social–ecological systems, which focuses on revealing the mechanisms of interaction among system elements, together with China’s decision-oriented research on natural carrying capacity (NCC), jointly constitute two important paradigms of sustainability research. This paper provides a systematic review of the progress of decision-oriented NCC research in China. The research began with single-factor carrying capacity assessments of land and water resources, focusing on how many people these resources could support and informing early population and resource management policies. Later, in response to the complex spatial planning demands posed by post-Wenchuan earthquake reconstruction, NCC research evolved into a comprehensive assessment system integrating resources, environmental conditions, ecological factors, and disaster risks, addressing challenges such as functional zoning, cross-scale application, and dynamic assessment, and ultimately becoming a foundational task of territorial spatial planning. On this basis, methods for territorial function suitability assessment and regional sustainability early warning were further developed, substantially strengthening NCC’s role in evidence-based decision-making. Although the interactions among elements within the Earth’s surface system remain incompletely understood, China’s NCC research has established a distinctive and viable pathway for decision-oriented NCC assessment. It also outlines future directions for deepening understanding of the system’s dynamic mechanisms and bridging regional and global sustainability boundaries.

## Teaser

A critical review and original analysis of China’s decision-oriented natural carrying capacity research reveals a distinctive pathway linking evidence-based spatial governance to broader sustainability research and enabling sustained progress in the field.

## INTRODUCTION

**Figure 1.**
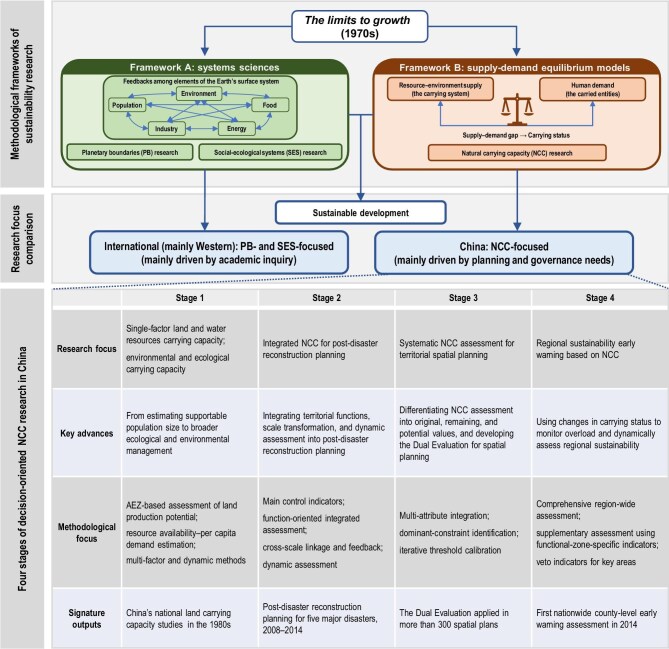
NCC methodological frameworks and the four development stages of decision-oriented NCC research in China.

Sustainability has long been a central concern for researchers and policymakers worldwide. Since the 1970s, the publication of *The Limits to Growth* has established both a foundational academic framework and a paradigmatic philosophy for sustainable development [1]. Building on this foundation, two distinct methodological frameworks have emerged and continue to shape research at both global and local scales (Fig. 1).

The first framework, rooted in systems science, characterizes the driving forces and feedback mechanisms among key Earth system elements to understand the sustainability dynamics of the Earth’s surface system, including population, food, industry, energy, and the environment. This tradition underpins contemporary research on planetary boundaries [2–4] at the global scale and social–ecological systems (SES) at the regional scale [5–7]. These approaches have garnered significant academic attention for their rigorous mathematical representation of element interactions and their focus on fundamental questions regarding the Earth system’s resilience [8,9]. The second framework builds on the concept of land resource carrying capacity. Drawing on supply–demand equilibrium models from economics, this framework conceptualizes resources and the environment as the ‘carrying system’ and human activities as the ‘carried entities’. It then examines the balance between environmental supply and human demand, thereby turning the complex problem of sustainability into a measurable question of the supply–demand gap and using overload as a criterion for assessing sustainability [10–12]. The strength of this approach lies in its ability to capture the essence of sustainability through the carrying relationship between these two subsystems, even when scientific understanding of the complex interactions within the Earth’s surface system remains incomplete. Consequently, this framework has attracted attention not only in academia but, more importantly, in decision-making practice.

Since the 1970s, the international academic community largely led by Western scholars has predominantly adopted the first framework, particularly in studies on planetary boundaries and regional social–ecological systems. In planetary boundaries research, scholars are expanding from biophysical threshold measurements toward an integrated ‘safe and just’ perspective [13], while seeking to downscale global boundaries to the national level to explore trade-offs between social well-being and environmental limits [14]. In regional social–ecological systems (SES) research, scholars widely employ the Coupled Human and Natural Systems and telecoupling frameworks to analyze non-linear feedback mechanisms [6,15] and systemic risks triggered by climatic and ecological tipping points [5]. In contrast, Chinese research has largely developed around the second framework, particularly in studies of regional resource and environmental carrying capacity, while the first framework has also been applied in regional SES research. In the former, carrying capacity assessment has been deeply integrated into territorial spatial planning practice in China [16]. The research paradigm has evolved from single-factor assessments of land and water resources to comprehensive assessments of integrated natural–economic–social systems, focusing on quantifying the dynamic balance between resource–environment supply and human demand and identifying key constraints [17]. In regional SES research, the cascade effects of major national ecological programs have been examined through the ‘pattern–process–service–well-being’ pathway, particularly in ecologically fragile regions such as the Loess Plateau [18].

Despite these advances, two critical gaps remain. First, studies that systematically link global planetary boundaries with regional SES and resource and environmental carrying capacity research are still rare. Existing work has mainly focused on downscaling global biophysical boundaries identified by planetary boundaries research and using them as boundary conditions for regional environmental footprints [19]. Second, across these areas of research, including planetary boundaries, SES, and carrying capacity, studies that support decision-making for sustainability at global, national, or local scales tend to attract sustained attention and drive continued research progress [2,6,20].

China has faced persistent pressures on sustainable development during four decades of rapid growth. As a result, both regional SES and regional natural carrying capacity (NCC) research have received increasing attention in China and have become increasingly oriented toward decision-making applications. This decision-oriented focus is a distinctive feature of Chinese research in these areas. Notably, China’s series of decision-oriented studies on NCC have made innovative contributions to global sustainability research. This paper provides a systematic review of China’s decision-oriented research on NCC across four stages of development. It examines advances in theory and methodology, changes in China’s carrying capacity status, and the role of NCC in supporting decision-making. Furthermore, it also discusses how future regional NCC research can draw on the interaction-focused paradigms of SES and planetary boundaries research, as well as the links between regional NCC and planetary boundaries.

## RESOURCE CARRYING CAPACITY RESEARCH WITH ‘HOW MANY PEOPLE CAN BE SUPPORTED’ AS THE STARTING POINT FOR APPLICATION

The size of population that can be sustained ultimately depends on food production, which in turn relies on the productive capacity of land, particularly cropland. This idea is consistent with the concept of the carrying capacity proposed by Bentley in the late 19th century based on livestock studies in pastoral regions [21,22]. The world food crisis in 1972 sparked widespread debate over whether population growth had exceeded the Earth’s carrying capacity limits. In response to these concerns, the Food and Agriculture Organization of the United Nations (FAO) conducted studies estimating how many people land resources could support, providing a scientific basis for international food aid programs [11]. These approaches were subsequently introduced to China and extended to single-factor water resources carrying capacity studies, providing scientific support for the implementation of the family planning policy. This illustrates how policy and management needs renewed interest in the carrying capacity concept nearly a century after it was first proposed. Later, the incorporation of environmental and ecological carrying capacity marked a major shift in carrying capacity research, expanded it from single-factor assessment to multi-factor analyses, from relatively simple to more complex ones, and from estimating ‘how many people can be supported’ to supporting broader fields such as ecological and environmental management and planning.

### Land resource carrying capacity research

An influential line of resource carrying capacity research in China emerged in the 1980s with studies examining how many people the country’s land resources could support [11]. From 1949 to 1979, China’s population grew from 542 million to 975 million, while per capita cropland area decreased from 1807 to 1020 m²/person [23]. During this period, China’s limited land resources had to support not only grain production to feed a rapidly growing population but also cotton cultivation to meet clothing needs in the absence of a developed chemical fiber industry. As a result, pressure on land resources intensified.

To address the question of how many people China’s land resources could support, Chinese researchers adopted the Agro-Ecological Zoning (AEZ) method developed in FAO studies on the potential population carrying capacity of land and conducted two large-scale land carrying capacity assessments [24–26]. The theoretical origins of the AEZ method can be traced to the biological equation proposed in *Road to Survival* [27] and to British studies estimating the population carrying capacity of colonial lands [28]. In this approach, land resource carrying capacity is defined as the population that can be supported by grain production or available cropland at a given point in time. To account for future dynamics, the AEZ method incorporates grain production capacity under different technological scenarios and population nutritional requirements at different stages of development as key parameters to project future population carrying capacity.

Research on how many people land resources can support establishes relationships between cropland area, grain yield under different technological conditions, nutritional requirements at different stages of development, and population size. In essence, such studies of relatively simple subsystems of the Earth’s surface system can be analyzed using the system dynamics approach within the first framework of sustainability research. The main source of uncertainty in these assessments lies in estimating grain yield per unit area under future technological conditions. The State Land Administration and Shi Yulin estimated that China’s land resources could support populations of 1.37 billion and 1.28 billion by 2000, respectively, with the difference mainly resulting from different assumptions about cropland yield levels, with estimates of 3887.7 kg/ha by the State Land Administration and 3570 kg/ha by Shi Yulin (Table 1). In reality, China’s population in 2000 reached 1.27 billion, exceeding Shi Yulin’s predicted value of 1.23 billion. This discrepancy was largely due to technological progress, which increased grain output and effectively raised the actual carrying capacity. The growth in grain production during this period was mainly driven by large-scale use of chemical fertilizers and pesticides. Over the nearly 30 years since 1990, China’s grain yield per unit area has increased by 45.79%, while chemical fertilizer and pesticide use has increased by 102.71% and 79.17%, respectively [23]. Although chemical fertilizer and pesticide applications have increased land grain output in the short term, their long-term effects, such as soil structure degradation, water eutrophication, and other forms of agricultural non-point source pollution, have reduced land carrying capacity. This highlights that while technological progress can enhance carrying capacity in the process of transforming nature, attention must also be paid to its negative effects, which has important implications for regional sustainability.

**Table 1.** Representative studies on land carrying potential and population carrying capacity in China.

					Population carrying capacity (billion people)				
Researcher	Forecast period	Arable area (million ha)	Input level	Nutritional level	Calculated by per capita calorie intake and per capita protein intake	Calculated by per capita grain consumption	Actual population (billion people)	Grain yield per unit area (kg/ha)	Per capita grain crops (kg/person)
**State Land Administration 1989–1994**	Current term (1989)	125.23	Low	2662 kcal calorie intake and 69.7 g protein intake per capita	1.14	—	1.13	3089.70	402.60
	Medium-term (2000)	122.18	Medium	2720 kcal calorie intake and 74 g protein intake per capita	1.40	—	1.27	3887.70	448.10
	Long-term (2050)	121.67	High	2800 kcal calorie intake and 82 g protein intake per capita	1.74	—	—	4828.65	458.60
**Shi 1985–1992**	Current term (1985)	132.77	Low	375 kg annual grain consumption per capita	—	1.05	1.06	2640.00	375.00
	Medium-term (2000)	126.51	Medium	400 kg annual grain consumption per capita	—	1.23	1.27	3570.00	400.00
	Long-term (2025)	123.51	High	450 kg annual grain consumption per capita	—	1.55	—	4920.00	450.00

In this study, arable area, nutrient level, grain yield per unit area, per capita grain crops, and population carrying capacity are given for each study forecast period. The input level follows the definition of FAO. Low input level assumes no fertilizer and pesticide applications and no soil conservation measures; medium input level assumes the use of improved hand tools and draught implements, the application of some fertilizers and pesticides, some simple soil conservation measures to mitigate the loss of productivity from land degradation, and the cultivation of a mixture of crops currently being grown and the most calorie and protein productive crops; high input level assumes complete mechanization, full use of optimum genetic material, necessary farm chemicals and soil conservation measures, and the cultivation of only the most calorie and protein productive crops. Data are derived from Refs. [24,25].

### Water resources carrying capacity research

Following single-factor land carrying capacity research, water resources carrying capacity became another major direction of single-factor resource carrying capacity research as water scarcity emerged as an increasingly important constraint on global sustainability. In 1989, Falkenmark first proposed the water stress index, using annual renewable freshwater availability per capita to characterize the degree of regional water scarcity [29]. In 1997, the United Nations first assessed the water stress faced by human societies and found that approximately one-third of the world’s population lived in countries experiencing moderate to high water stress, a share that could rise to two-thirds by 2025. These findings underscored the severity of the global water crisis [30]. In the same year, the United Nations Commission on Sustainable Development launched the Global Freshwater Initiative, helping to advance international research on the sustainable use of water resources [31]. Since then, research on single-factor water resources carrying capacity has gradually expanded to cover the full chain of water demand, supply, and management, as well as multiple water types, such as surface water, groundwater, and virtual water, and multiple dimensions, including water resources, water environment, and water ecology [32,33].

In line with global trends, water scarcity has become a key factor affecting China’s sustainable development [34]. In 2023, China’s per capita freshwater availability is about 1747.47 cubic meters, only around one quarter of the global average. Moreover, 38.32% of surface water resources are distributed in the southwestern mountainous region, where plains account for only 11.26% of the national total, whereas the northwestern region, covering 35.11% of the country’s land area, contains only 9.71% of surface water resources, indicating an extremely uneven spatial distribution [23]. Together with water-use conflicts between industry and agriculture, between urban and rural areas, and the rapidly growing demand for ecological water use, these conditions have intensified the constraints imposed by water resources on population and economic activities [35,36]. China’s water resources carrying capacity assessments have mainly focused on estimating the population that water resources can support. In 1981, Song Zicheng used mathematical models to estimate that China’s water resources could support a maximum population of only 630–650 million after a century [37]. In 1998, Yuan Jianhua used trend extrapolation to estimate that when China reached the level of a middle-income country, the maximum population supported by China’s water resource would be about 1.15 billion [38]. Notably, both national-scale estimates based on water resources were lower than those based on land resources in Section *Land Resource Carrying Capacity Research*, suggesting that water resources constitute a stronger constraint factor than land resources in China’s NCC. Research on single-factor water resources carrying capacity has largely followed the methodological approach used in land resource carrying capacity studies, using total resource availability and per capita demand as key parameters to estimate the supportable population size. However, because water resources are characterized by complex hydrological process, mobility, cyclicity, and diverse utilization pathways, water resources carrying capacity research has incorporated approaches such as system dynamics and virtual water analysis, providing analytical support for multi-objective decision-making involving industrial and agricultural development and urbanization [39–41].

### Environmental carrying capacity and ecological carrying capacity research

Environmental and ecological issues are also key factors affecting sustainability across different spatial scales. Research on environmental and ecological carrying capacity has driven a shift from single-factor studies of land resource carrying capacity and water resources carrying capacity to integrated multi-factor research. At the same time, the focus of application has shifted from estimating ‘how many people can be supported’ to broader fields such as ecological and environmental management and planning.

As research data and methods expanded, studies on environmental and ecological carrying capacity developed rapidly during this period. Traditional single-factor resource carrying capacity research focused primarily on the supply capacity of individual natural resources such as land and water, whereas environmental and ecological carrying capacity research expanded to include the pollutant assimilative capacity of environmental media such as air, water bodies, and soil, as well as the comprehensive supporting capacity of ecosystems [42]. Environmental carrying capacity research examines the maximum pollutant load that environmental systems can accommodate under specific conditions and time periods, while ecological carrying capacity research emphasizes the overall maintenance capacity of ecosystems, seeking to assess both the demand of human activities on natural ecosystems and the capacity of ecosystems to sustain those activities [43].

Compared with single-factor resource carrying capacity studies, research on environmental and ecological carrying capacity has undergone three major shifts. First, a shift from single-factor to multi-factor research. Single-factor resource carrying capacity studies focus only on the capacity of a specific resource, whereas environmental and ecological carrying capacity research requires not only assessing individual factors but also integrating multiple factors to evaluate overall system carrying capacity [44]. By introducing methods such as energy analysis and ecological footprint, this research has converted different types of resource consumption and environmental use into standardized units, thereby addressing the problem of unit consistency in multi-factor integration [45]. Second, a shift from simple system studies to complex system studies. Single-factor resource carrying capacity research is largely based on static calculations and is therefore insufficient to capture the complex and heterogeneous nature of environmental and ecological factors. To address the complex feedback mechanisms among different components of the system, approaches such as the DPSIR framework and system dynamics have been widely adopted. These approaches have enabled a transition from static accounting to dynamic integrated simulation, improving the ability to analyze system evolution processes [46,47]. Third, a shift from estimating ‘how many people can be supported’ to research aimed at supporting ecological and environmental management and planning. Environmental carrying capacity assessments are mainly used to support environmental management mechanisms such as total pollutant load control and pollutant discharge permits, providing a quantitative basis for pollution control. Ecological carrying capacity research, by contrast, focuses on evaluating regional ecological surplus or deficit conditions, providing a scientific basis for delineating ecological protection boundaries and maintaining ecosystem health [48].

## TOWARD INTEGRATED ASSESSMENT: NCC RESEARCH IN SUPPORT OF POST-DISASTER RECONSTRUCTION PLANNING

Adjusting population control policies based on single-factor carrying capacity assessments of land or water resources represents a straightforward decision-making application. Similarly, optimizing environmental and ecological management and planning policies through research on environmental carrying capacity and ecological carrying capacity constitutes a sector-specific decision-making application for sustainable development. However, these approaches remain limited in addressing the broader issues of comprehensive spatial organization for regional sustainable development at the territorial level. In fact, in pursuing sustainable development at the global, national, or local scale, using resource and environmental carrying capacity to inform the functional orientation and spatial configuration of different regions, optimize the allocation of resource and environmental factors across regions, and guide the transformation and upgrading of short-term and long-term development strategies constitutes an important set of decision-making applications for achieving the sustainable development goals (SDGs) [10]. The key scientific challenges in NCC research underlying these critical applications include whether different land-use types and territorial functions can be assessed within a common NCC assessment system, how scale linkage and effective transmission can be achieved in NCC assessment across regional and local spatial scales, and how dynamic assessment can be conducted as the Earth’s surface system and corresponding NCC continue to change. The demand for NCC research generated by post-disaster reconstruction planning after the 2008 Wenchuan Earthquake encompassed these key scientific questions and critical application needs, thereby driving major advances in integrated NCC research. Between 2008 and 2014, China experienced four additional major natural disasters [49–52]. The continuously refined NCC research methods addressed a series of challenges, including delineating reconstruction functional zones, precisely estimating reasonable population size, conducting fine-scale assessment through spatial downscaling, and developing dynamic assessment frameworks to address uncertainty. These efforts demonstrated the applicability of NCC assessment in formulating reconstruction strategies and preparing spatial plans. NCC assessment was subsequently designated by the central government as a foundational task in China’s territorial spatial planning, thereby opening a pathway for science-based decision-making in implementing China’s sustainable development strategy.

### Estimating population capacity based on territorial functions

In 2008, an *M*s 8.0 earthquake struck Wenchuan, leaving nearly 100 000 people dead or missing and affecting an area of approximately 132 400 km^2^. As in many less-developed regions across China, the Wenchuan area had long lacked rational spatial planning. Disordered population distribution and land-use patterns substantially increased exposure to disaster risks, thereby amplifying the losses caused by natural hazards (Appendix S1, Figs S1 and S2, and Table S1). The primary issues in reconstruction planning were the delineation of reconstruction functional zones and the determination of reasonable population size in different areas. These constituted an integrated carrying capacity assessment problem that could not be addressed by earlier single-factor resource assessments or by multi-factor ecological and environmental carrying capacity assessments.

The Earth’s surface system is a territorial system in which different subsystems and regions perform distinct territorial functions, such as urbanization, food production, and ecological security functions, under the overarching goal of sustainable development. The structural configuration of elements within the natural geographic environment influences the formation and evolution of these territorial functions. Establishing the interrelationships between territorial functions and the structural configuration of natural elements forms the foundation for research on integrated NCC. Accordingly, integrated NCC refers to the integration of sustainability-related attributes, including resources, environment, ecology, and disaster risk, associated with natural elements such as water, land, atmosphere, and biota [53]. The indicator system for assessing integrated NCC varies according to the specific territorial function. In the post-Wenchuan Earthquake reconstruction planning, three territorial function types were delineated for the entire affected area based on integrated NCC assessment, namely urban reconstruction zones, agricultural and rural zones, and ecological protection zones [49].

**Figure 2.**
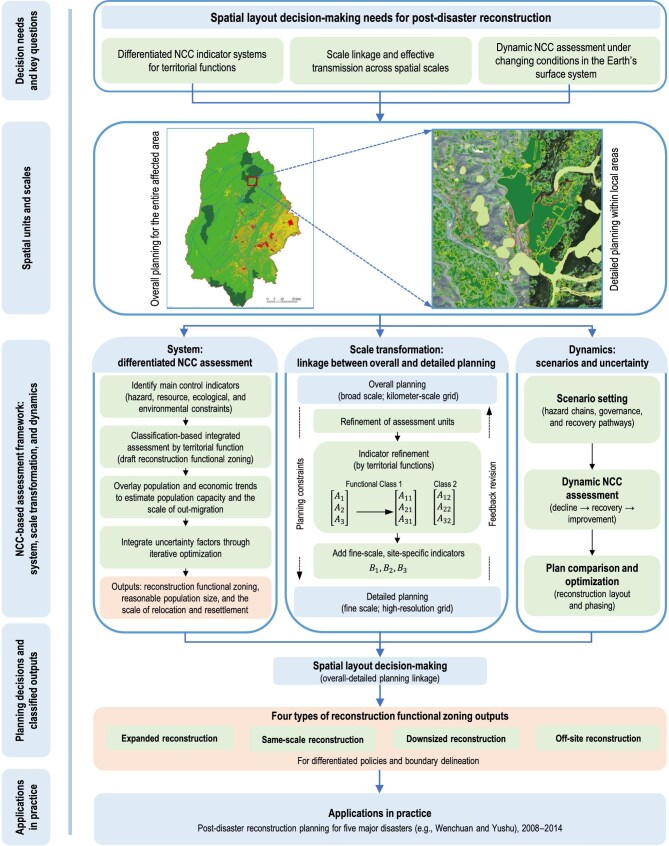
Schematic diagram of NCC-based multi-level spatial planning linkage for post-disaster reconstruction.

Subsequently, integrated NCC assessment was used to support territorial function zoning, site selection for towns and villages, and the optimization of population distribution in the post-disaster reconstruction for Wenchuan (Fig. 2). Compared with earlier carrying capacity assessment, NCC assessment in the Wenchuan reconstruction had to address more complex decision-making problems. Its focus expanded from estimating the population size of the carried entities to optimizing land-use functional zoning and site selection for towns and villages within the carrying system, and from calculating maximum carrying capacity thresholds to supporting carrying capacity-oriented decisions on population distribution [49]. Given the characteristics of the earthquake-affected area, the severity of earthquake damage and the hazard level of secondary mountain hazards were selected as the main control indicators, and the assessment was carried out in four steps. Step 1: Assessment of the main control indicators. Areas falling within the highest-risk range of these indicators were directly designated as ecological land, while areas with relatively high safety levels were retained as candidate sites for towns and villages. Step 2: Classification-based integrated assessment oriented toward territorial functions and the delineation of reconstruction functional zones, including ecological protection, agricultural production, and urban development, different indicator sets were identified for classification-based carrying capacity assessment, resulting in a draft reconstruction functional zoning scheme at the grid-cell scale. Step 3: Analysis of local population characteristics, economic development conditions, and future development prospects to determine the spatial pattern of population distribution. By comparing these results with the pre-disaster population distribution and the spatial distribution of disaster-induced casualties, the scale of the population requiring relocation from different reconstruction functional zones was estimated [54]. Step 4: Taking into account the uncertainty factors discussed below and the refined assessment results obtained through spatial downscaling, the final assessment outputs were produced through iterative optimization to inform decision-making, including reconstruction functional zones, reasonable population size, and the scale of population relocation and resettlement (Fig. S3 and Table S2).

### Scale transformation of NCC to meet the needs of multi-level spatial planning

Reconstruction planning includes both overall planning and detailed planning for different functional zones. Different levels of reconstruction planning require different classifications of land-use functions within the region and corresponding indicator systems for assessment. As planning proceeds to progressively finer scales, assessment becomes increasingly refined. Accordingly, NCC assessment needs to adopt methods for scale transformation to meet the requirements of different levels of reconstruction planning [51].

At the broad scale of overall planning for the entire affected area, territorial functions are mainly classified into macro categories such as urban, agricultural, and ecological zones. For example, urbanized areas may be categorized into administrative centers, tourism towns, and agro-processing towns, while ecological zones may be divided into different types of protected natural areas. By contrast, at the finer scale of detailed planning within functional zones, urban areas need to be further subdivided into specific land parcels such as residential areas and public service facility areas. Major agricultural production areas need to be further differentiated into rural construction land and agricultural production land parcels. Ecological zones also need to be subdivided into core zones, buffer zones, and other categories based on ecological vulnerability. As planning is downscaled, functional classification becomes increasingly refined, evaluation units shift from kilometer-scale grids to higher-resolution grids suited to fine-scale assessment, and the indicators system, assessment precision, and assessment methods also need to be adjusted accordingly. For example, in the fine-scale NCC assessment of reconstruction functional zones for towns and villages, remote sensing interpretation and regional-scale model-based analysis conducted at the broad scale of overall planning are insufficient to identify localized geological hazards and site conditions. They are therefore inadequate for supporting small-scale construction decisions, such as the site selection of different types of construction land, the delineation of control boundaries and ranges, and the determination of disaster mitigation standards for buildings [55,56]. Accordingly, fine-scale assessment for detailed planning builds on higher-resolution topographic and resource and environmental data and incorporates analyses that are meaningful at finer scales, including secondary mountain hazards, engineering geology, and hydrogeology. Combined with field damage surveys and on-site investigation data, such as micro-scale investigations of geological hazard points and simulations of flood inundation ranges in small watersheds, these assessments make it possible to evaluate mor precisely the severity and spatial extent of specific hazards. This in turn supports the delineation of disaster avoidance zones for towns and villages and the determination of disaster mitigation standards, including seismic resistance requirements for reconstruction [57]. During the downscaling assessment process, the preliminary reconstruction scheme derived from large-scale overall planning based on NCC assessment is first assigned to small-scale detailed planning as a planning constraint. The results of local fine-scale NCC assessment are then fed back into the large-scale assessment, and where inconsistencies arise between results at different scales, the preliminary reconstruction scheme is revised accordingly. Through repeated iterative validation, this process produces a spatial configuration scheme in which the regional overall plan and local parcels are consistent with the results of NCC assessment. Ultimately, reconstruction planning classified reconstruction functional zones for town and village development into four types, namely expanded reconstruction, same-scale reconstruction, downsized reconstruction, and off-site reconstruction. This provided a more targeted basis for the formulation of overall and detailed post-disaster reconstruction plans, as well as reconstruction strategies and differentiated policies.

### Dynamic assessment of NCC in response to post-disaster reconstruction uncertainty

Severe natural disasters can sharply reduce the NCC of affected areas within a very short period, whereas post-disaster reconstruction gradually enhances NCC during the reconstruction process. During reconstruction, some changes in the elements of the carrying system remain uncertain. Based on simulations of inundation ranges resulting from barrier lake breaches, simulations of changes in the risk of secondary hazards under different aftershock scenarios, and dynamic assessments of carrying capacity recovery under different mitigation scenarios, a dynamic assessment model was developed to accommodate reconstruction uncertainty. This model can support comparisons among multiple reconstruction schemes and the long-term optimization of reconstruction planning.

## SYSTEMATIC NCC RESEARCH IN SUPPORT OF SPATIAL PLANNING

Since NCC research addresses the differentiation of territorial functions among regions and the assessment of natural conditions across different spatial scales, spatial planning is its most suitable decision-making application. Based on the role of NCC in post-disaster reconstruction, the central government designated NCC assessment as a foundational task in China’s spatial planning, thereby advancing NCC research into a new stage of system research. System research on NCC has involved two major innovations. First, to accommodate the dynamic changes in NCC, and with a focus on the carrying system, NCC assessment has been differentiated into assessments of original value, remaining value, and potential value, providing an effective approach to dynamic NCC assessment [53]. Second, given that the formation of future territorial functions and changes in the development and protection patterns of the Earth’s surface system are influenced not only by NCC but also by other factors, an integrated assessment method has been developed that takes both NCC and these additional factors into account, referred to as territorial function suitability (TFS) assessment [58]. These two innovations have substantially improved the capacity of NCC research to diagnose the rationality of current spatial utilization and to inform the planning of future spatial utilization patterns, thereby strengthening its role in supporting spatial planning applications.

### Assessment of the original, remaining, and potential values of NCC in China

**Figure 3.**
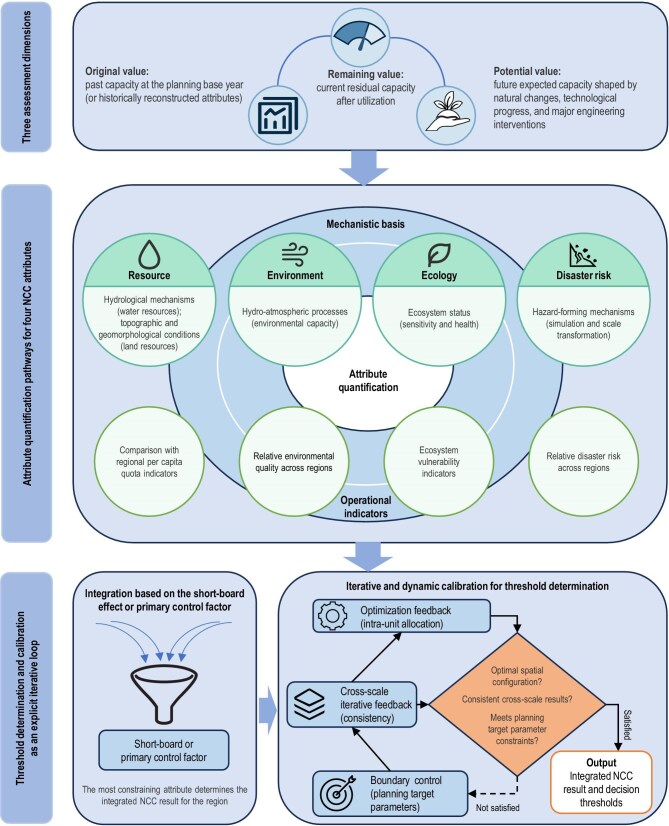
Methodological framework for integrated NCC assessment: dimensions, attribute quantification, and iterative threshold calibration.

Integrated NCC assessment refers to the quantitative integration of the sustainability-related attributes of the carrying system, namely resource, ecological, environmental, and disaster-related attributes associated with natural elements. It comprises three assessment dimensions, namely original value, remaining value, and potential value [53]. The original value refers to the capacity of the natural system at a given point in the past to support human activities through its water and land resources, environmental capacity, and ecosystem health, after avoiding natural disaster risks (Fig. 3). In practice, given data availability, the planning base year is usually selected as the reference point. The origin value of NCC is then represented by either directly observable attribute values of natural elements in the base year or historical attribute values reconstructed based on the mechanisms governing the formation of those elements. The remaining value refers to the residual capacity of the current natural system to support human activities. In practice, the current attribute values of natural elements are generally taken as the remaining value of NCC. The potential value refers to the carrying capacity expected to be formed at a future point in time as a result of natural changes, major interventions in nature, and human activities.

In integrated NCC assessment, the quantification of sustainability-related attributes of natural elements draws primarily on methods developed in earlier single-factor assessments of land resource carrying capacity and water resources carrying capacity, environmental carrying capacity, and ecological carrying capacity. These methods include calculating available water resources based on hydrological mechanisms, estimating available land resources based on topographic and geomorphological conditions, determining environmental capacity based on hydro-atmospheric processes, evaluating ecological sensitivity based on ecosystem status, and assessing natural disaster risks based on principles of structural geology and natural hazard science [59,60]. However, in quantifying the attributes of natural elements, the determination of thresholds remains a persistent challenge. In theory, threshold values for individual attributes could be identified on the basis of changes in the system’s carrying status. In practice, however, the scientific mechanisms governing the interactions among elements and their impacts on the system have not been fully clarified, making it difficult to calculate such thresholds directly using explicit mathematical models. To meet the needs of planning applications, the quantitative assessment of attributes usually relies on several alternative approaches. Resource attributes can be quantified by comparing land, water, and other resource elements with regional per capita quota indicators. Environmental attributes are often quantified using the relative level of environmental quality across regions as a proxy, because the scientific mechanisms underlying environmental capacity remain unclear. Ecological attributes can be quantified directly using indicators of ecosystem health, such as ecosystem vulnerability, because research on ecological carrying capacity is relatively well developed. Disaster attributes are quantified mainly on the basis of the micro-scale hazard-forming mechanisms of disaster-inducing factors and are the upscaled, through model simulation and scale transformation, to obtain the relative level of disaster risk across regions. When the quantified attribute values of natural elements are integrated to derive the result of integrated NCC assessment, the short-board effect or primary control factor method is usually adopted. In other words, the quantified result of the attribute exerting the strongest constraint among the sustainability-related attributes of natural elements is taken as the integrated NCC assessment result for the region. Although mechanistic integration based on analytically derived structural parameters describing interactions among elements would be a more precise approach, its practical application remains limited because the interaction mechanisms among elements within the system are still not fully understood.

To ensure effective linkage between NCC assessment results and management decision-making, the determination of thresholds for element attributes often requires repeated iteration and dynamic calibration [59,60]. In practice, this is usually achieved through three main approaches. The first is optimization feedback based on the allocation of elements within a regional unit. By using the matching relationships among elements within the unit, such as the matching between land and water resources, iterative optimization is carried out to improve the spatial allocation of resource and environmental elements. The second is iterative feedback across multiple scales. To address differences in natural elements and data precision requirements across spatial scales in NCC assessment, cross-scale iterative mechanisms are established to ensure consistency between assessment results at broader and finer scales. The third is boundary control based on planning target parameters. This is the most critical step in the reasonable determination of NCC assessment thresholds for decision-making applications. For example, the built-up area defined in a planning scheme can be used as the upper limit for the expansion of construction land, while the grain self-sufficiency rate can be used as the lower bound for cropland retention. These parameters serve as key constraints in the final determination of reasonable NCC thresholds. In decision-making applications, this approach transforms threshold determination into a structured problem of comparing and ranking attribute values under the constraints of planning target parameters, thereby providing a workable pathway for addressing an otherwise intractable scientific problem. This has become one of the major contributions of China’s NCC research. Even so, it should be recognized that the iterative calibration process described above remains a technical means of approximation. A fundamental solution to NCC threshold delineation and the determination of classification schemes still depends on a thorough clarification of the intrinsic coupling mechanisms among natural elements.

**Figure 4.**
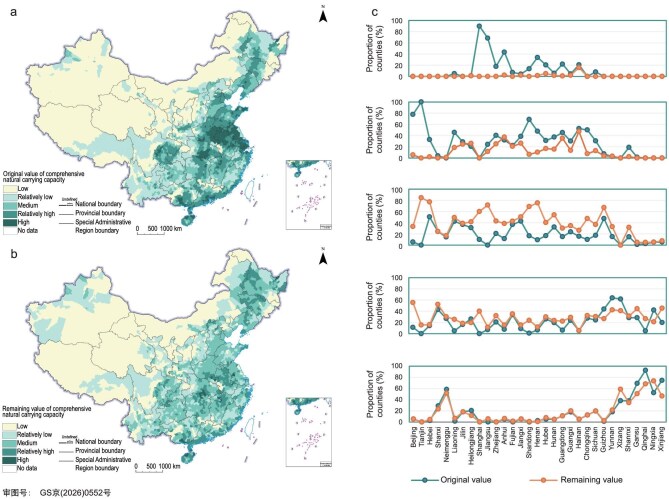
Evaluation results of original and remaining values of comprehensive NCC in China. (a) Original value of comprehensive carrying capacity. (b) Remaining value of comprehensive carrying capacity. (c) Proportion of counties within different NCC levels by province.

Using the above methods, this study selected 2010 as the base year for original value assessment and 2020 as the reference year for remaining value assessment. It assessed China’s available water and land resources, environmental quality, ecosystem vulnerability and importance, and natural disaster risks, and integrated these results to obtain the original and remaining values of China’s integrated NCC (Fig. 4 and Tables S3 and S4). Comparison of the two assessments revealed three main characteristics of the spatial pattern of China’s integrated NCC. First, the plains and basins along the major rivers in China’s eastern monsoon region were mainly classified as areas of relatively high or high of NCC, including the Northeast China Plain, the North China Plain, the middle and lower reaches of the Yangtze River Plain, the Sichuan Basin, and the Pearl River Delta. By contrast, the arid and semi-arid regions in the northwestern non-monsoon zone and the Tibetan Plateau were mainly classified as areas of relatively low or low NCC. Second, the areas with high integrated NCC in China were also those where decline was most pronounced. Third, the loss of available land resources and the deterioration of environmental quality were the main drivers of the decline in China’s integrated NCC. In the southeastern coastal region, the decline was driven mainly by the loss of available land resources. In the Sichuan Basin of southwestern China, it was attributable primarily to deteriorating environmental quality. In the North China Plain, both factors exerted pressure. The analysis for China shows that, when integrated NCC assessment is used to support decision-making applications, its results can provide an important basis for assessing the effectiveness of plan implementation.

Prediction of the potential value of NCC should focus on three main sources of change. The first is the effect of climate change on the spatial patterns of water resources carrying capacity and ecological carrying capacity, such as the influence of the trend toward warmer and wetter conditions in the arid regions of northwestern China on water resources carrying capacity. The second is the effect of technological progress on the intensity of resource consumption, pollutant emissions, and the capacity to withstand risk. These changes in turn affect assessment results in which carrying capacity is expressed in terms of the scale of the carried entities. The third is the positive and negative effects of major engineering interventions on NCC. For example, at broad scales, inter-basin water transfer may be undertaken to alleviate regional water shortages [61]. At intermediate scales, environmental management measures may be implemented to restore or expand environmental capacity [62]. At fine scales, land consolidation and hazard prevention measures may be used to reduce disaster risk [63]. However, the effects of such interventions on NCC are often uncertain, as in the case of large inter-basin water transfer projects, mountain cutting and lake filling to expand land resources, and dams constructed to intercept debris flows. The prediction of the potential value of NCC has a clear dual role in spatial planning. On the one hand, it serves as a rigid constraint and boundary condition for planning, because predicted potential values are used to determine the scale and layout of future development. On the other hand, it is itself a direct object of planning intervention and protection, because ecological restoration or engineering measures may be used proactively to improve expected future NCC [64]. Planning practice shows that changes in the potential value of NCC display pronounced scale effects. At broad scales, natural geographical patterns tend to be relatively stable, and a single project usually has limited influence on the overall pattern of NCC. At fine scales, however, major engineering interventions can significantly reshape local NCC. More importantly, in decision-oriented practice, decision-makers often focus on the positive effects of engineering interventions on carrying capacity, whereas scientific research should place greater emphasis on demonstrating the potential negative effects of artificially reshaping nature. Prediction of the potential value of NCC should therefore go beyond engineering feasibility alone. It should clarify the ecological safety boundaries associated with engineering interventions, establish scientifically grounded risk early warning mechanisms, and provide decision-makers with clear warnings about ecological limits and risks.

### Research on territorial function suitability and its comparison with NCC research in spatial planning applications

An overreliance on NCC assessment results in spatial governance may lead decision-makers into the trap of environmental determinism [65]. Therefore, addressing the linkage between NCC assessment and its application in spatial planning has become a major direction of innovative research to enhance the value of NCC assessment as a foundational task in territorial spatial planning. At the national scale, the first task of spatial planning is to allocate land uses according to local conditions. Different land uses correspond to the different territorial functions that regions perform in achieving sustainable development. Urban areas concentrate population and economic activity, agricultural areas ensure food security, and ecological areas provide ecological security buffers. Rational planning the spatial structure of these three territorial functions, together with the spatial organization within each of them, is essential for achieving an orderly spatial structure and sustainable development in China [53]. In addition to constructing indicator systems for NCC assessment according to the formation conditions of the three territorial functions, the TFS index derived from an extended model based on NCC provides a more direct indicator for decision-making applications in territorial planning and spatial governance:


(1) \begin{eqnarray*}
{\mathrm{TFS}} = K \times {\mathrm{NCC}}.
\end{eqnarray*}

**Figure 5.**
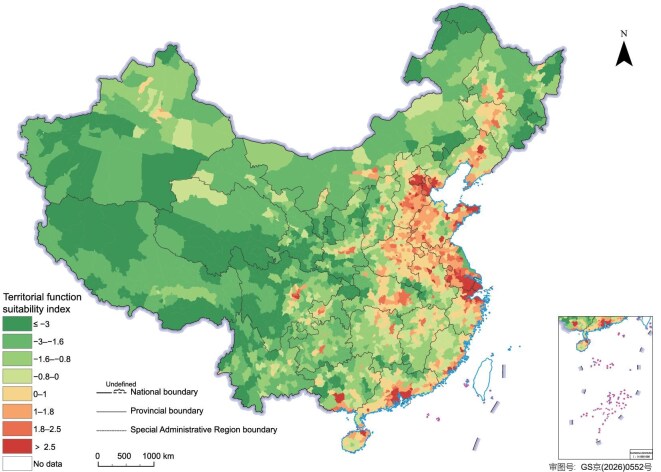
Comprehensive evaluation of territorial function suitability with a development orientation in China. Adapted from Ref. [58].

In this equation, *K* is a function of geographical location and system integrity and is highly flexibility. It may represent either the full set or a subset of the factors that give rise to the differences between the zoning pattern of China’s three major territorial functions and the distribution pattern of NCC. In the national TFS assessment, *K* is represented by an integrated set of indicators, including the capacity to agglomerate population and economic activity, the integrity of ecosystems and major food production bases, transport accessibility and locational advantage, and the degree of alignment with national strategic priorities [53]. Figure 5 presents the classification map of China’s TFS index derived from a development-oriented assessment.

**Figure 6.**
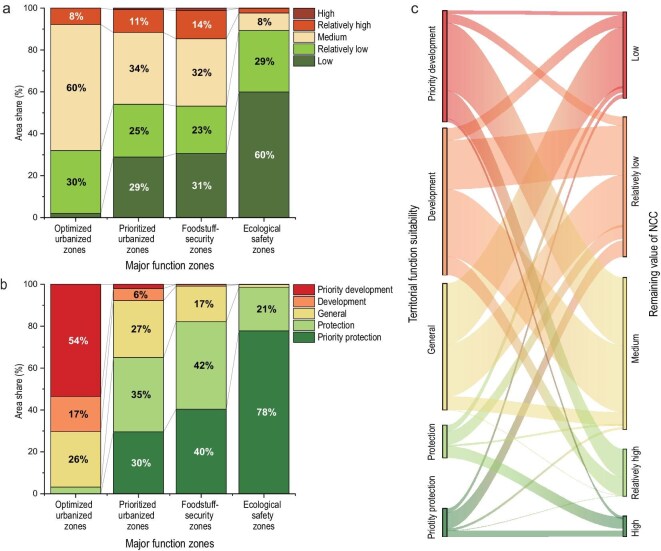
Distribution and conversion of levels for the remaining value of comprehensive NCC and the territorial function suitability in different major function zones. (a) Distribution of levels for the remaining value of comprehensive NCC in different major function zones. (b) Distribution of levels for the territorial function suitability in different major function zones. (c) Conversion from NCC to territorial function suitability assessment. The remaining value of the comprehensive NCC is used in this research. See Fig. S4 for China’s Major Function Zoning.

NCC assessment and TFS assessment together constitute the foundational task supporting different types of spatial planning in China. They are collectively referred to as the Dual Evaluation and have been applied in more than 300 spatial plans at the national, provincial, and municipal levels. A comparison between China’s Major Function Zoning scheme with the results of the Dual Evaluation shows that the TFS assessment results fit the planning scheme better than the NCC assessment results do, indicating that extended NCC research has significantly strengthened its capacity to support applications (Fig. 6).

### Major progress in China’s research on carrying capacity at the watershed and regional scales

In addition to nationwide studies on NCC, research on NCC at the watershed and regional scales has also made important contributions to sustainable development at the watershed and regional levels. Given the marked differences in geographical environments and stages of development across China, the NCC challenges faced by different regions vary considerably, and research on NCC at the watershed and regional scales exhibits strong regional characteristics.

Based on water scarcity, the arid and semi-arid regions of northwest China are characterized by relatively low original, remaining, and potential NCC values. Water resources have consistently been the primary factor constraining urbanization, industrialization, and agricultural modernization, making this region a focal area for regional NCC research in China. As a result, NCC research in northwest China has mainly focused on the conflicts and trade-offs between water use and other functions, such as ecological conservation, regional development, and sustainable livelihoods. It has also examined ecological water demand to avoid overexploitation of water resources and ways to enhance land carrying capacity by expanding cultivated land through water diversion. Already urbanized areas, by contrast, are often characterized by high original NCC values but insufficient remaining values. In these areas, interactions between human and natural systems are more complex and intense, and NCC significantly constrains the development of urban agglomerations. Research has therefore focused on identifying and alleviating limiting factors in order to enhance the potential value of NCC, assessing the carrying status of urban agglomerations, and exploring the rational allocation of NCC among different areas and sectors within urban agglomerations. Fang Chuanglin’s study of the coupling and coordination between the environmental and socioeconomic sustainability systems in the Beijing–Tianjin–Hebei urban agglomeration shows that environmental carrying factors are key targets for the future sustainable regulation of urban agglomerations [66]. Research on the Pearl River Delta urban agglomeration, by contrast, has focused on the impacts of land development on NCC and the related optimization strategies [67].

The Tibetan Plateau and the Loess Plateau, whose natural systems are highly distinctive both with China and worldwide, have long been focal areas of NCC research. The effects of critical ecological issues and ecosystem fragility on NCC, the evolving relationship between intensifying human activities and natural ecosystems, and green pathways to sustainable development have consistently been central themes of NCC research in these regions [68]. With the expansion of tourism development on the Tibetan Plateau, NCC research has gradually shifted from the traditional issues of pastoral overloading to the ecological risks associated with growing non-local populations [69]. In addition, the integrated assessment of resource and environmental carrying capacity in Tibet led by Feng Zhiming, together with Fan Jie’s exploration of spatial carrying capacity for the Earth’s Third Pole national park cluster, has opened up new research areas [70,71]. The latter incorporates social carrying capacity and infrastructure carrying capacity together with NCC into the assessment framework and attempts to provide a scientific basis for estimating the reasonable capacity of the national park cluster on the Tibetan Plateau. In the Loess Plateau, early research in NCC-related fields focused on soil erosion control and strategies for enhancing regional NCC. In recent years, studies led by Fu Bojie have increasingly adopted social–ecological system frameworks aligned with international research to examine the relationships among ecological carrying capacity [72], water and land resource use, food production, and rural livelihood improvement. A key scientific issue is the trade-off between afforestation and water resources carrying capacity, particularly in relation to soil moisture depletion. These findings have been applied in regional ecological assessment and have become an important basis for guiding spatial development in the region [73].

River basins are relatively integrated ecological systems and natural geographical units, making them ideal for analyzing water resources, a highly mobile element within the carrying system. Research has mainly focused on the interactions among mountains, rivers, forests, farmland, lakes, and grasslands within basin-scale NCC, on the scientific basis for the rational allocation of carrying capacity between upstream and downstream areas and among main streams and tributaries, and on how to coordinate overall ecological protection with socioeconomic development across the basin and enhance the basin’s capacity for sustainable development. In research on the Yellow River Basin, ecological footprint and water footprint approaches have been used to assess NCC characteristics in areas along the river. These assessments have become an important basis for delineating ecological compensation zones within the basin [74]. Life cycle assessment has also been used to evaluate the carrying constraints associated with regional socioeconomic activities across their entire life cycle, thereby informing sustainable basin management under climate change. In the Yangtze River Basin, changes in the habitats of endemic and endangered species, especially fish, together with biodiversity, have been used as key indicators for assessing basin-scale NCC [75]. A representative contribution of NCC research in the Heihe River Basin led by Cheng Guodong is that it addressed the threat posed by excessive water exploitation in the upper and middle reaches of this inland arid river basin to downstream oasis systems [41]. In this context, ecological water transfers and assessments of their ecological and socioeconomic benefits have become an effective means of balancing the water demands of socioeconomic and natural systems. Islands, like river basins, are also relatively independent and integrated territorial units. In addition to the widely studied issue of freshwater scarcity, recent research has also paid increasing attention to technical pathways such as expanding land resource carrying capacity through land reclamation and alleviating water shortages through seawater desalination [76].

## EXTENDED NCC RESEARCH IN SUPPORT OF REGIONAL SUSTAINABILITY EARLY WARNING

While NCC research provides a foundational basis for planning, using regional sustainability early warning to dynamically assess plan implementation represents an important extension of NCC research. During regional development, the carrying status formed between the carrying system and the carried entities changes continuously, and once overload occurs, the region is judged to be unsustainable [20]. The basic principle of this line of research is to use changes in carrying status to support the monitoring and early warning of regional development sustainability. China began applying NCC to sustainability early warning research in 2014, and the resulting findings have provided timely policy support for curbing unsustainable development in overloaded regions.

The complexity of regional sustainability makes direct modeling of sustainability early warning difficult. Using NCC-based carrying status assessment to support sustainability early warning is a relatively feasible methodological path [77,78]. There are typically four equilibrium relationships between the carrying system and the carried entities, namely non-overload, critical overload, overload, and an irreversible state. Regional sustainability early warning can be developed by tracking changes in the characteristic values associated with these relationships during the regional development process, and this approach requires attention to three methodological points. It requires a comprehensive assessment covering the entire region and basic elements related to water, land, the environment, and ecology under scenarios in which technological progress and management policies dynamically regulate resource use efficiency and pollution emission intensity. It also requires supplementary assessment using specific indicators for different functional zones, such as haze pollution levels in urbanized areas and changes in cultivated land area in major agricultural production zones. In addition, for key areas where natural factors play a dominant role, such as the core zones of nature reserves, certain early warning indicators should be treated as veto criteria. For example, water quality should be used as a veto indicator in drinking water source areas. If substandard water quality is detected, the area should be classified directly as overloaded, regardless of the carrying status of other elements of the carrying system. On the basis of these assessments, regional sustainability early warning results can be generated, and adaptive policies can then be proposed in response to the causes of overload, including resource and environmental remediation, functional zone development and management, and optimization of human production and daily living activities.

**Figure 7.**
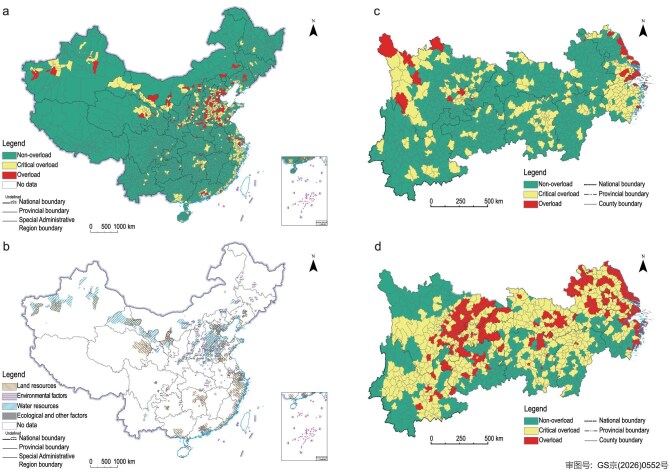
The results of early-warning assessments of regional sustainability in China. (a) National assessment for China conducted in 2014. (b) Spatial distribution of key constraint factors within overloaded and critical overload zones. (c) Regional sustainability early-warning for the YREB in 2017 based on the 2014 methodology. (d) Regional sustainability early-warning for the YREB in 2017 based on the updated methodology incorporating a more comprehensive set of pollution indicators. Data are derived from Refs. [20,79].

In 2014, the first nationwide county-level assessment of regional sustainability early warning showed that areas classified as overloaded or critically overloaded accounted for 11.26% of China’s land area, but contained 44.13% of the national population and 56.44% of GDP [20,79]. China’s major economic belts and urbanized regions thus faced more severe unsustainability pressures. Two elements of the carrying system were the main causes of overload (Fig. 7 and Tables S5 and S6). One was water resources, with water resource overload accounting for 73.58% of all overloaded areas and occurring mainly in the arid and semi-arid water-scarce regions of northwest China. The other was environment, with 84.29% of the population in overloaded areas living in areas overloaded in environmental terms. China’s coastal regions and urbanized areas, where economic development started earlier, were the main areas of environmental overload, while the peripheral areas of central cities and the zones linking urban agglomerations had generally entered a state of critical overload. In addition, among the 197 overloaded counties, 86 exceeded the thresholds for the haze-specific indicator. These findings suggest that both long-standing and emerging environmental pollution problems, together with water scarcity, have become major challenges that China must address in pursuing modernization. Enhancing water resources carrying capacity and environmental carrying capacity is therefore an important pathway to achieve sustainable development in China.

A prominent methodological issue in successive early warning assessments of regional sustainability is that changes in the indicator system often lead to different warning results. This can be seen from simulation assessments of sustainability in the Yangtze River Economic Belt (YREB) using the 2014 and 2017 versions of the indicator system. When the 2014 indicator system was applied, the 2017 assessment showed only a slight aggravation of overload compared with 2014, with the number of overloaded counties increasing from 23 to 35. In the 2014 indicator system, the environmental indicators were limited to exceedance of environmental carrying capacity for SO_2_ and COD. However, in the 2017 assessment, the environmental indicators were expanded to include multiple environmental pollutant concentration indicators, including inhalable particulate matter (PM_2.5_ and PM₁₀), ozone, carbon monoxide, biochemical oxygen demand, total phosphorus, and total nitrogen, and the overload situation became much more severe than in 2014. The number of overloaded counties increased from 23 to 194, and their share rose sharply from 2.54% to 71.58%. The main distribution of overloaded counties also expanded westward from the coastal Yangtze River Delta to inland areas, including the Wuhan metropolitan area and Chengdu–Chongqing metropolitan area. These results show that when new pollutants or environmental pressure indicators are introduced into regional sustainability early warning assessment, the results often change substantially, and their spatial pattern also shifts markedly. A deeper understanding of the factors affecting NCC can enable early warning systems to reflect changes in regional sustainability more accurately. Therefore, the indicator system should be dynamically adjusted and refined to better capture newly emerging resource and environmental constraints and risk factors.

## DISCUSSION AND CONCLUSION

### Conceptual framework and methodological evolution of decision-oriented NCC research

**Figure 8.**
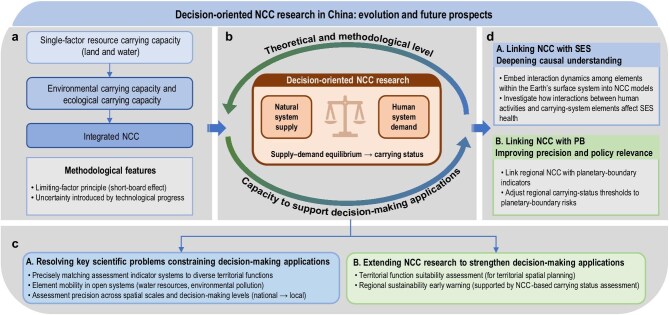
Decision-oriented NCC research in China. (a) Conceptual evolution of NCC from single-factor to integrated assessment. (b) A virtuous interaction between NCC research and decision-making applications. (c) Two core characteristics driving the development of decision-oriented NCC research. (d) Future prospects for decision-oriented natural carrying capacity research.

Natural carrying capacity research originated in biological inquiries into how many livestock a pasture could support. It subsequently developed into single-factor assessments of land resource carrying capacity and water resources carrying capacity aimed at determining how many people could be supported, then gradually extended to assessments of environmental carrying capacity and ecological carrying capacity, and further to integrated carrying capacity assessment covering sustainability-related issues concerning resources, ecology, environment, and disasters. In this process (Fig. 8), its basic methodological characteristics mainly include two aspects. One is the limiting-factor principle, that is, the threshold of a key constraining indicator is used to represent the overall threshold of the system’s carrying status. The other is attention to technological progress and to the uncertainty it introduces into NCC assessment. It should be noted that, unlike decision-oriented NCC research, NCC research conducted mainly for academic purposes more often explores the carrying status of regional systems through different combinations of a limited number of elements. Examples include water–energy–food (WEF) nexus research, which analyzes the constraints and trade-offs among key resource elements in production and consumption, and climate–land–water system research, which examines how the matching relationship between land use and key resource elements evolve under climate change [80]. Such research usually focuses on explaining interactions among elements and the underlying scientific mechanisms, whereas decision-oriented NCC research focuses on quantitatively characterizing the carrying status of regional systems by identifying the supply–demand equilibrium between the two subsystems of natural system supply and human system demand.

The development of decision-oriented NCC research is mainly characterized by two aspects. First, it focuses on resolving key scientific problems that constrain decision-making applications. These problems mainly include the differentiated requirements for precisely matching assessment indicator systems to the diverse functions of the Earth’s surface system, the complex effects of the mobility of elements such as water resources and environmental pollution on regional NCC assessment in open regional systems, and the effects of different requirements for assessment precision across spatial scales and decision-making levels from the national to the local level [81]. Resolving these problems has become a major driving force in the continued development of decision-oriented NCC research.

Second, it takes NCC research as its foundation and improves the usefulness of assessment results for decision-making through research extension. For example, territorial spatial suitability assessment for territorial spatial planning at different levels, and NCC monitoring and early warning assessment serving dynamic regional sustainability early warning, both extend NCC research and thereby strengthen its capacity to support decision-making applications. In applied research such as monitoring and early warning, the practical demand for constructing highly timely resource and environment indicator systems has driven data collection and network development for space–air–ground integrated monitoring systems, while also promoting innovative research in early warning methods and model algorithms.

Overall, decision-oriented NCC research still starts from the most fundamental scientific questions of carrying capacity and is jointly driven by basic scientific principles and decision-making needs to develop new theories and methods. In this process, it takes the resolution of key scientific problems that limit decision-making applications as an important goal. This has improved both the theoretical and methodological levels of NCC research and its capacity to support decision-making applications, thereby promoting a positive interaction between NCC research and decision-making applications.

### Future prospects for decision-oriented NCC research

Enhancing the capacity of NCC to support decision-making applications ultimately depends on progress in foundational NCC research itself. In China, compared with the strong progress of decision-oriented carrying capacity research, research on the dynamic mechanisms among internal elements within the global and regional systems of the Earth’s surface system, as well as on how these interactions affect planetary boundaries and the health of regional social–ecological systems, remains relatively underdeveloped. In the future, NCC research should make breakthrough progress in two dimensions.

First, decision-oriented NCC research should not be limited to the study of the equilibrium relationship between the carrying system and the carried entities. Rather than continuing to focus mainly on carrying status assessment, future research should further deepen the study of underlying causal mechanisms and actively advance research on the dynamics of the terrestrial component of the Earth’s surface system. This requires incorporating into NCC models the system dynamics of interactions among elements within the Earth’s surface system formed by the natural environment and human activities, and examining how different elements of the carrying system directly and indirectly affect the carrying status of different elements of the carried entities. More importantly, it requires investigating changes in the overall health of social–ecological systems resulting from interactions between human activities and the different elements of the carrying system, so as to characterize more accurately the internal mechanisms underlying changes in NCC. In this way, decision-oriented NCC research can provide more precise scientific solutions for coordinating humans and nature and enhancing NCC on the basis of causal understanding.

Second, unlike the above dimension, which focuses on system dynamics to clarify the scientific mechanisms of carrying capacity, research on the link between regional NCC and planetary boundaries is more concerned with improving the precision of carrying status assessment and the policy relevance of NCC research. In planetary boundary research, seven of the nine boundaries have been transgressed. This reflects the cumulative effects of regional overload on global boundary outcomes, and the relationship between the two is evident. We tentatively suggest that, for indicators whose planetary boundaries have already been transgressed or are at risk of transgression, stricter controls should be imposed at the regional level. This is an important pathway for coordinating regional and global sustainability. For NCC research, this means linking regional NCC with planetary boundary indicators and adjusting the thresholds for regional carrying status according to the transgression status of planetary boundary indicators, the rate of change, the degree of transgression, and the likelihood of future transgression. The more severe the planetary boundary transgression, the stricter the corresponding regional thresholds should be. Accordingly, developing an integrated indicator framework and conducting coordinated research linking global planetary boundaries, regional social–ecological systems, and regional NCC should become an important for scientists at both global and local levels in advancing the SDGs.

## Supplementary Material

nwag205_Supplemental_File
