# Supplementary material for: China’s natural carrying capacity: its change and progress of decision-oriented study
Source: Natl Sci Rev. 2026 Apr 2;13(9):nwag205. doi: 10.1093/nsr/nwag205 (PMC13196708; doi:10.1093/nsr/nwag205)
Supplement: nwag205_Supplemental_File [file nwag205_supplemental_file.docx]

**Supplementary information**

**China's natural carrying capacity: its change and progress of decision-oriented studies**

**Supplemental Appendix 1.** **Estimation of the magnification effect of population exposure on natural disaster losses due to irrational spatial planning.**

One of the biggest reasons for the huge death toll is that a large proportion of the population lives near inhospitable earthquake fault due to unscientific planning, resulting in a large increase in exposure. In order to test the contribution of excessive exposure due to unscientific planning to disaster losses, we introduce the evaluation results of territorial functional suitability (Evaluation on the type and scale of territorial space adaptation development based on land, water, environment, disasters, population gathering capacity, development status of urban built-up areas, economic development level, which is used to guide the layout of industries and populations in China from 2011) [1].

According to the evaluation results of territorial functional suitability, of the 37 counties in the core affected area of the Wenchuan earthquake, 17 are urbanization areas, 7 are major agricultural production areas and 13 are key ecological function areas. Based on the population data in 2007, the population and land development intensity of urbanization areas remain unchanged. The intensity of land development in major agricultural production areas is set at 40% of that in urbanization areas, and that in key ecological function areas is set at 15% of that in urbanization areas [1]. Then, according to the ratio of population and land development intensity, the population numbers of main agricultural production areas and key ecological function areas are re-fitted as the number of simulated populations.

In order to ensure the consistency of vulnerability during simulation, GDP per capita, percentage of population with age above 15 and under 65, percentage of male residents, percentage of employed, and number of medical institutions per km^2^ in 2007 are selected to construct vulnerability indicators [2-4]. The number of deaths is taken as the parent node, and the number of simulated populations, seismic intensity and the above vulnerability indicators are taken as the child nodes to construct the Bayesian network [5]. Based on the actual number of deaths of each county in Wenchuan earthquake, the conditional probability of each node is determined by Expectation-Maximization algorithm [6]. Finally, on the basis of the given conditional probability, the possible death toll of each county subjected to the Wenchuan earthquake intensity under the simulated population is calculated. The socioeconomic data is derived from the National Bureau of Statistics of China.

***Methods***

**Structure of Bayesian network.** Bayesian network (BN) is a complete model of the system of interest, including its component variables and the probabilistic relationships between them [5]. To construct a BN, the variables of indicators should first be identified. The number of deaths, which is assumed to be a parent of exposure-, vulnerability- and hazard- related indicators, is the root node. Exposure-related indicator is the number of simulated populations according to the evaluation results of territorial functional suitability [1], hazard-related indicator is the seismic intensity, vulnerability-related indicators include GDP per capita, percentage of population with age above 15 and under 65, percentage of male residents, percentage of employed, and number of medical institutions per km^2^ [2-4].

**Determining the conditional probability.** A conditional probability measures the probability of an event given that another event has occurred. Once a BN framework is constructed, the conditional probability of each node given their parent nodes should be determined, that is, the conditional probability of an exposure-, vulnerability-, or hazard-related indicator given a number of deaths should be determined (equation 3). Based on the actual number of deaths of each county in Wenchuan earthquake, the conditional probability of each node is determined by Expectation-Maximization algorithm [6]. Here, the number of deaths and exposure-, vulnerability-, or hazard-related indicators all divided into five states (Supplemental Table 1).

 (1)

Where, *L_i_* represent the *i* state of the number of deaths *L*, *i=1,2,…,5*, and *v_kj_* represents the *j* state of exposure-, vulnerability-, or hazard-related indicator *k*, *k=1,2, …,7, j=1, 2,…,5.*

**The number of deaths calculation.** Based on the posteriori probability of the target the number of deaths obtained above, when the states of all exposure-, vulnerability-, and hazard-related indicator are given as *j*, the probability of the number of deaths *L_i_* occurring can be calculated (equation 4).

 (2)

Where, *L_i_* represents the *i* state of the number of deaths *L*, *i=1,2,…,5*, and

*v_kj_* represents the *j* state of exposure-, vulnerability-, or hazard-related indicator *k*, *k=1,2, …,7*.

Then the number of deaths, with given all exposure-, vulnerability-, and hazard-related indicators states *j*, can be calculated as equation (5).

 (3)

Where, *L_imean_* represents the mean value in each *L* state, *L_1mean_*=5, *L_2mean_*=50, *L_3mean_*=500, *L_4mean_*=5,000, *L_5mean_*=1/2(10%+ Maximum death toll at county level in Wenchuan earthquake), and *P(L_i_)* is the corresponding probability of the target *L_i_* occurring.

The number of deaths with other states of exposure-, vulnerability-, and hazard-related indicator can be calculated in the same way.


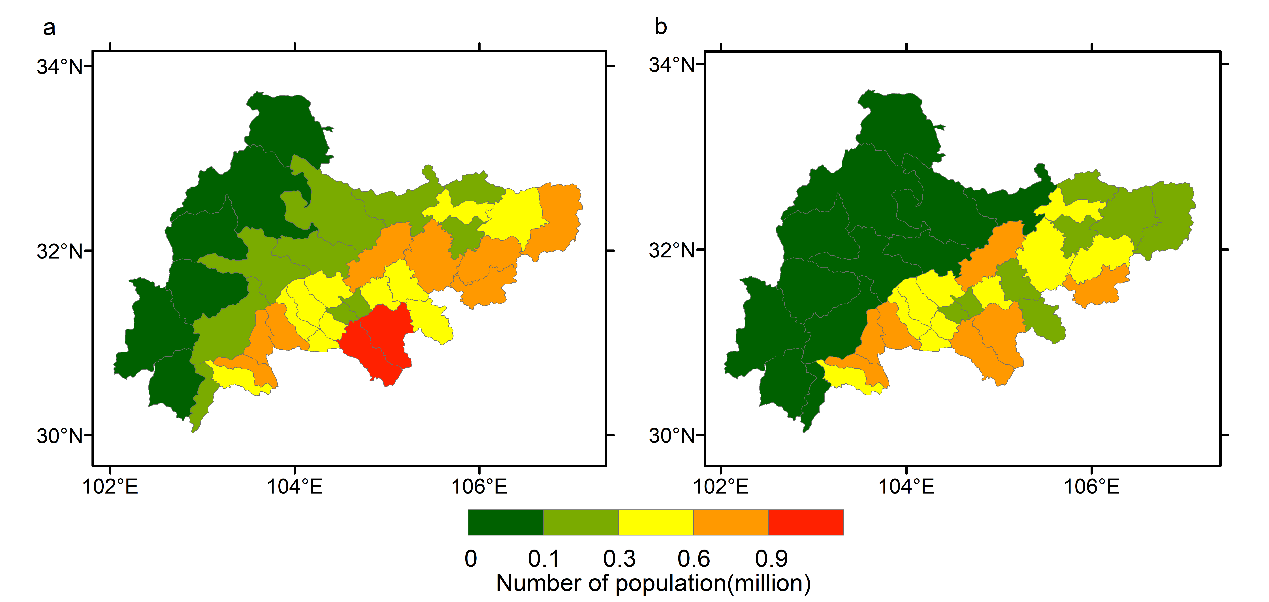


**Supplemental Fig. 1. Distribution of** **population size during the 2008 Wenchuan earthquake.** (a) Actual population size. (b) Simulated population size under scientific planning. The actual population of the core area of the Wenchuan earthquake was 16.41 million, and the simulated population is 12.19 million, which only accounted for 74.28% of the actual population. Among the five counties affected by the XI intensity, Pengzhou and Dujiangyan are urbanization area, and the simulated population is consistent with the actual population. Wenchuan, Beichuan and Pingwu are key ecological function area. The actual population of these three counties are all between 0.1 to 0.3 million, while the simulated population are less than 0.1million. The total simulated population of the three counties fell by nearly 70 percent compared with the actual population. The socioeconomic data is derived from the National Bureau of Statistics of China. See Supplemental Appendix 1 for the evaluation methods.


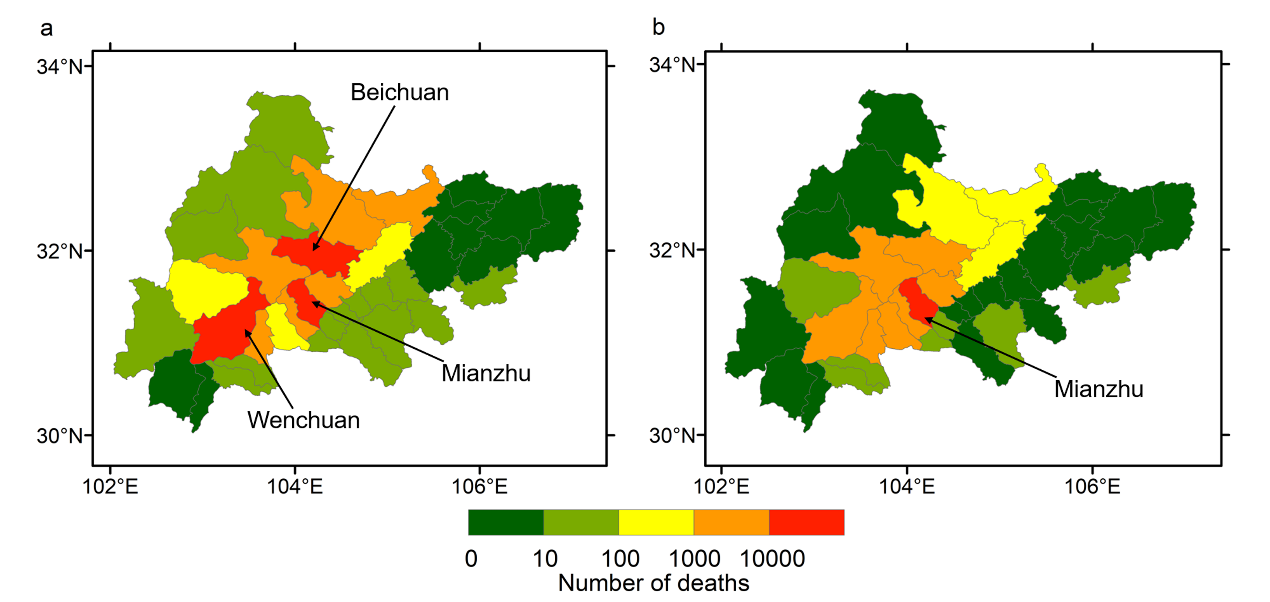


**Supplemental Fig. 2. Distribution of** **death toll during the 2008 Wenchuan earthquake.** (a) Actual death toll. (b) Simulated death toll under scientific planning. In the 17 urbanization counties whose total population has not been adjusted, the simulated death toll is basically consistent with the actual death toll, so it can be basically verified that the simulation results of the model can fully reflect the actual losses of the disaster. The actual death toll was 77,530 and the simulated death toll is only 39,901. Hence, with the same vulnerability and hazard intensity, increased exposure due to unscientific planning nearly doubled the death toll in the Wenchuan earthquake. The number of counties with more than 10,000 deaths drops from three (Wenchuan, Mianzhu, Beichuan) to one (Mianzhu). Mianzhu is an urbanized area, the simulated population is consistent with the actual population, thus the simulated death is basically consistent with the actual death. In Wenchuan and Beichuan, the number of simulated deaths decreased significantly compared with the actual number of deaths due to the reduction of simulated population. The socioeconomic data is derived from the National Bureau of Statistics of China. See Supplemental Appendix 1 for the evaluation methods.


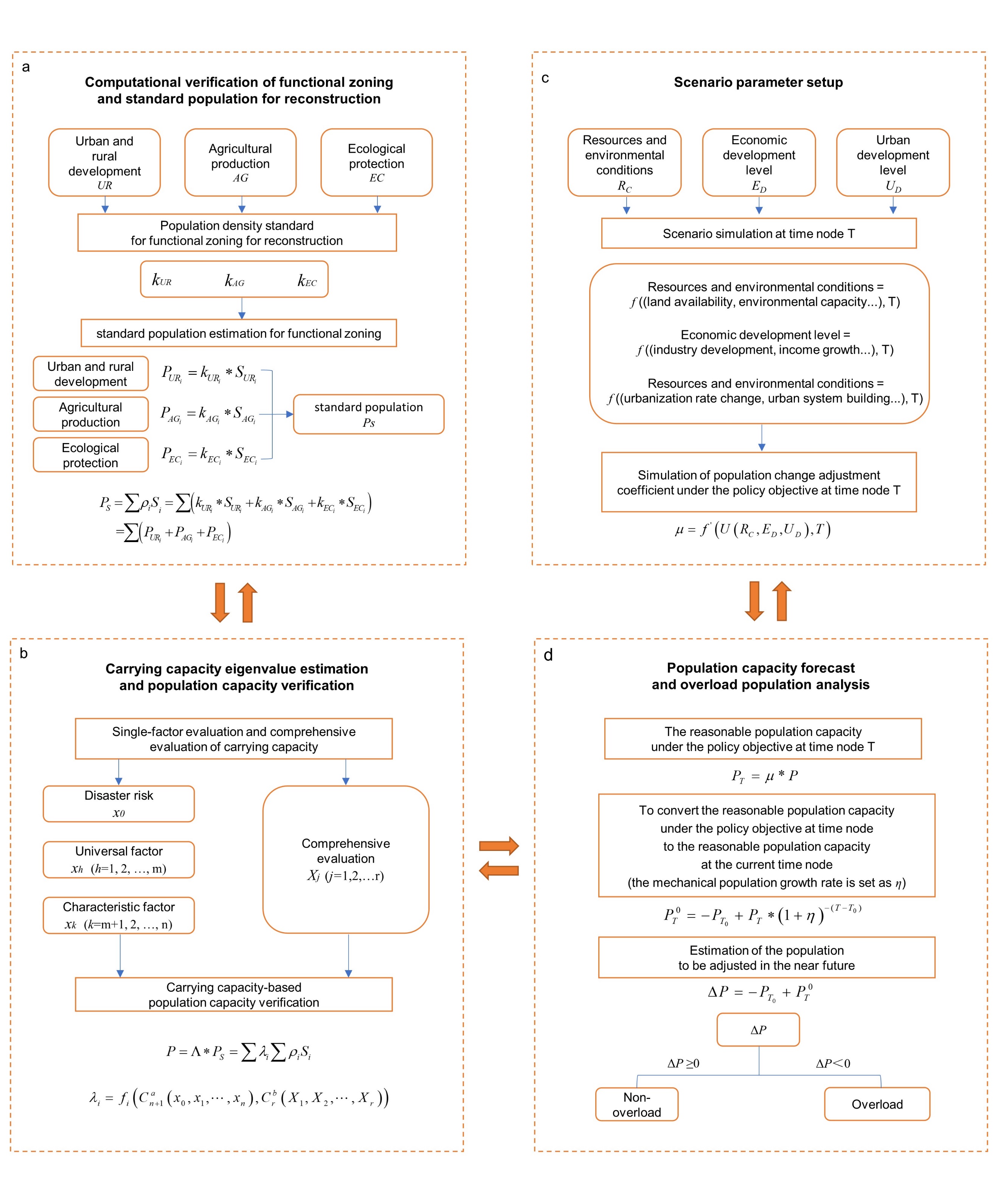


**Supplemental Fig. 3. Technical roadmap of reasonable population capacity estimation for post-disaster restoration and reconstruction.** The technical roadmap of the reasonable population capacity estimation can be divided into four steps. (a) First, the preliminary population capacity is calculated based on the population density criteria in the different functional zones set by the planning and combined with the area in the different functional zones obtained from the evaluation. (b) ​Second, the population capacity calculated in different functional zones is revised based on the NCC evaluation results, and the adjustment coefficients are calculated based on the NCC. (c) Third, to set up future planning scenarios and calculate population capacity adjustment coefficients based on different planning scenarios. (d) Combined with the population capacity adjustment coefficient obtained before, the preliminary population capacity is revised. ​During the evaluation process, the population capacity needs to be repeatedly iterated and revised based on feedback from the fine evaluation results to the large-scale evaluation results to obtain the final population capacity evaluation results.​


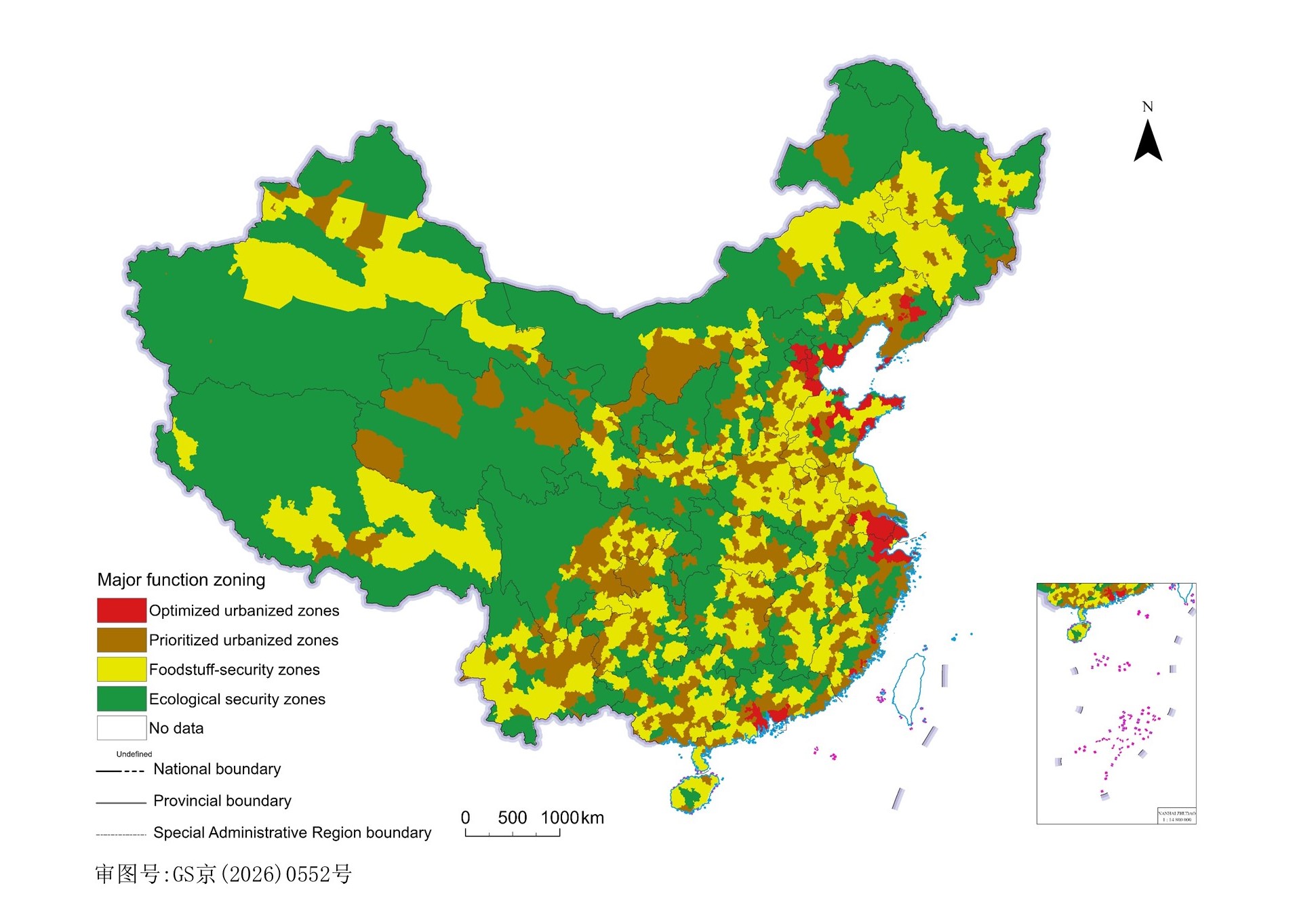


**Supplemental Fig. 4.** **Major Function Zoning in China.** China's Major Function Zoning divides China's territorial land space into four types: optimized urbanized zones, prioritized urbanized zones, foodstuff-security zones, and ecological security zones. To date, the Major Function Zoning has been the most influential spatial planning in China. Adapted from REF [7].

**Supplemental Table 1**

**Different states of indicators in Bayesian network.** The seismic intensity is the average intensity by county, which is calculated by the area of each county.

| **Indicator** | **States** |
| --- | --- |
| Number of simulated population (P) | P_1_<0.1 million  0.1 million≤P_2_<0.3 million  0.3 million≤P_3_<0.6 million  0.6 million≤P_4_<0.9 million  P_5_≥0.9 million |
| GDP per capita (G) | G_1_<6000 yuan  6000 yuan≤G_2_<10000 yuan  10000 yuan≤G_3_<14000 yuan  14000 yuan≤G_4_<18000 yuan  G_5_≥18000 yuan |
| Number of medical institutions per km^2^ (Mi) | Mi_1_<0.02 medical institutions/km^2^  0.02 medical institutions/km^2^≤Mi_2_<0.03 medical institutions/km^2^  0.03 medical institutions/km^2^≤Mi_3_<0.04 medical institutions/km^2^  0.04 medical institutions/km^2^≤Mi_4_<0.05 medical institutions/km^2^  Mi_5_≥0.05 medical institutions/km^2^ |
| Percentage of population with age above 15 and under 65 (Pa) | Pa_1_<72%  72%≤Pa_2_<73.5%  73.5%≤Pa_3_<75%  75%≤Pa_4_<76.5%  Pa_5_≥76.5% |
| Percentage of male residents (Ma) | Ma_1_<50%  50%≤Ma_2_<50.5%  50.5%≤Ma_3_<51%  51%≤Ma_4_<51.5%  Ma_5_≥51.5% |
| Percentage of employed (E) | E_1_<50%  50%≤E_2_<60%  60%≤E_3_<70%  70%≤E_4_<80%  E_5_≥80% |
| Seismic intensity (S) | S_1_<VII  VII≤S_2_<VIII  VIII≤S_3_<IX  IX≤S_4_<X  S_5_≥X |
| Number of deaths (L) | 0<L_1_<10  10≤L_2_<100  100≤L_3_<1000  1000≤L_4_<10000  L_5_≥10000 |

**Supplemental Table 2**

**Table template of population capacity evaluation.** In the evaluation of post-disaster emergency carrying capacity, the territorial functions of the reconstruction area are divided into three categories: urban and rural development, agricultural production, and ecological protection. In the process of population capacity evaluation, the disaster-hit areas and populations corresponding to the three functional areas will be counted respectively, and the population overload will be judged according to the reasonable population capacity that in the near future under different scenarios. See Supplemental Fig. 5 for specific evaluation process of reasonable population capacity.

| **Territorial functions** | | **Urban and rural development** | | | | | | | | | | **Agricultural production** | **Ecological protection** |
| --- | --- | --- | --- | --- | --- | --- | --- | --- | --- | --- | --- | --- | --- |
| Types of disaster-hit areas | | Statistics of counties and districts | | Disaster-affected population | | Reasonable population capacity | | | Overloaded population | | | … | … |
|  |  | Number | Area | Pre-disaster population | Post-disaster population | Scenario 1 | … | Scenario n | Scenario 1 | … | Scenario n | … | … |
| Hard-hit areas | Overload |  |  |  |  |  |  |  |  |  |  |  |  |
|  | Non-overload |  |  |  |  |  |  |  |  |  |  |  |  |
| Other disaster-hit areas | |  |  |  |  |  |  |  |  |  |  |  |  |
| **Sum** | |  |  |  |  |  |  |  |  |  |  |  |  |

**Supplemental Table 3**

**Evaluation results of original values of comprehensive NCC and single-factor NCC in China.** In this study, the single factors of NCC mainly include available land resources, available water resources, environmental capacity, ecological sensitivity, and natural disaster risk. See Supplemental Appendix 2 for general method for assessment of single-factor NCC. The assessment covers 2,378 districts and counties in China, excluding Hong Kong, Macao, Taiwan, and Xisha, Zhongsha, Dongsha, and Nansha islands, etc. The regional basic geographic data such as administrative divisions are derived from the National Geomatics Center of China. The population data is derived from the Tabulation on the 2010 Population Census of the People’s Republic of China by County. The 2010 GDP data is derived from the National Bureau of Statistics of China. The table is adapted from the REF [8].

| **Original values of NCC** | **Evaluation factor** | **Level of NCC** | **Number of units** | **Occupied land area** | | **Population in the area** | | **GDP in the area** | |
| --- | --- | --- | --- | --- | --- | --- | --- | --- | --- |
|  |  |  |  | Area (million km2) | Percentage (%) | Area (million person) | Percentage (%) | Area (trillion Chinese yuan) | Percentage (%) |
| Comprehensive NCC | Comprehensive NCC | Low | 459 | 4.16 | 53.33 | 104.36 | 7.83 | 2.35 | 11.55 |
|  |  | Relatively low | 547 | 1.42 | 21.29 | 176.82 | 13.27 | 3.21 | 21.36 |
|  |  | Medium | 498 | 1.07 | 11.41 | 251.47 | 18.88 | 6.00 | 19.10 |
|  |  | Relatively high | 634 | 1.52 | 10.42 | 516.49 | 38.77 | 13.26 | 21.73 |
|  |  | High | 240 | 1.17 | 3.55 | 282.99 | 21.24 | 8.80 | 26.26 |
| Water resource carrying capacity | Available water resources | Low | 451 | 4.16 | 44.58 | 143.04 | 10.74 | 3.88 | 11.55 |
|  |  | Relatively low | 444 | 1.42 | 15.22 | 253.52 | 19.03 | 7.18 | 21.36 |
|  |  | Medium | 373 | 1.07 | 11.41 | 247.17 | 18.55 | 6.42 | 19.10 |
|  |  | Relatively high | 589 | 1.52 | 16.29 | 358.54 | 26.91 | 7.30 | 21.73 |
|  |  | High | 519 | 1.17 | 12.50 | 329.87 | 24.76 | 8.83 | 26.26 |
| Land resource carrying capacity | Available land resources | Low | 594 | 4.44 | 47.49 | 133.66 | 10.03 | 2.34 | 6.97 |
|  |  | Relatively low | 574 | 2.71 | 29.02 | 240.41 | 18.05 | 5.09 | 15.14 |
|  |  | Medium | 408 | 0.99 | 10.62 | 289.69 | 21.75 | 7.81 | 23.23 |
|  |  | Relatively high | 323 | 0.57 | 6.08 | 260.16 | 19.53 | 7.76 | 23.09 |
|  |  | High | 479 | 0.63 | 6.78 | 408.22 | 30.64 | 10.61 | 31.57 |
| Ecological carrying capacity | Ecological sensitivity | Low | 278 | 2.24 | 23.99 | 70.22 | 5.27 | 1.22 | 3.62 |
|  |  | Relatively low | 360 | 2.79 | 29.90 | 121.08 | 9.09 | 2.22 | 6.60 |
|  |  | Medium | 868 | 3.02 | 32.32 | 383.24 | 28.77 | 8.49 | 25.25 |
|  |  | Relatively high | 485 | 0.82 | 8.83 | 392.56 | 29.47 | 10.77 | 32.03 |
|  |  | High | 384 | 0.46 | 4.96 | 365.03 | 27.40 | 10.93 | 32.50 |
| Natural disaster risk | Natural disaster risk | Low | 2 | 0.01 | 0.10 | 0.85 | 0.06 | 0.01 | 0.02 |
|  |  | Relatively low | 46 | 0.19 | 2.07 | 21.50 | 1.61 | 0.43 | 1.28 |
|  |  | Medium | 730 | 1.70 | 18.21 | 535.76 | 40.22 | 14.86 | 44.20 |
|  |  | Relatively high | 1330 | 4.82 | 51.56 | 657.00 | 49.32 | 15.63 | 46.50 |
|  |  | High | 267 | 2.62 | 28.05 | 117.02 | 8.78 | 2.69 | 8.01 |
| Sum | | | 2,378 | 9.34 | 100.00 | 1332.13 | 100.00 | 33.62 | 100.00 |

**Supplemental Table 4**

**Evaluation results of remaining values of comprehensive NCC and single-factor NCC in China.** In this study, the single factors of NCC mainly include available land resources, available water resources, environmental capacity, ecological sensitivity, and natural disaster risk. See Supplemental Appendix 2 for general method for assessment of single-factor NCC. The assessment covers 2,378 districts and counties in China, excluding Hong Kong, Macao, Taiwan, and Xisha, Zhongsha, Dongsha, and Nansha islands, etc. The regional basic geographic data such as administrative divisions are derived from the National Geomatics Center of China. The population data is derived from the Tabulation on the 2010 Population Census of the People’s Republic of China by County. The 2010 GDP data is derived from the National Bureau of Statistics of China. The table is adapted from the REF [9].

| **Remaining values of NCC** | **Evaluation factor** | **Level of NCC** | **Number of units** | **Occupied land area** | | **Population in the area** | | **GDP in the area** | |
| --- | --- | --- | --- | --- | --- | --- | --- | --- | --- |
|  |  |  |  | Area (million km2) | Percentage (%) | Area (million person) | Percentage (%) | Area (trillion Chinese yuan) | Percentage (%) |
| Comprehensive NCC | Comprehensive NCC | Low | 426 | 4.34 | 46.43 | 114.92 | 8.63 | 2.81 | 8.35 |
|  |  | Relatively low | 669 | 2.52 | 26.96 | 365.02 | 27.40 | 8.86 | 26.37 |
|  |  | Medium | 985 | 1.82 | 19.50 | 658.56 | 49.44 | 17.15 | 51.01 |
|  |  | Relatively high | 279 | 0.62 | 6.67 | 180.66 | 13.56 | 4.51 | 13.41 |
|  |  | High | 19 | 0.04 | 0.44 | 12.98 | 0.97 | 0.29 | 0.87 |
| Water resource carrying capacity | Available water resources | Low | 729 | 2.56 | 27.44 | 610.90 | 45.86 | 17.49 | 52.02 |
|  |  | Relatively low | 441 | 2.37 | 25.33 | 218.37 | 16.39 | 6.42 | 19.10 |
|  |  | Medium | 373 | 2.17 | 23.19 | 159.52 | 11.97 | 3.49 | 10.38 |
|  |  | Relatively high | 406 | 1.24 | 13.31 | 180.51 | 13.55 | 3.38 | 10.06 |
|  |  | High | 429 | 1.00 | 10.73 | 162.84 | 12.22 | 2.84 | 8.45 |
| Land resource carrying capacity | Available land resources | Low | 662 | 5.82 | 62.32 | 210.76 | 15.82 | 4.95 | 14.74 |
|  |  | Relatively low | 627 | 1.56 | 16.71 | 352.44 | 26.46 | 8.59 | 25.55 |
|  |  | Medium | 731 | 1.26 | 13.52 | 533.41 | 40.04 | 14.58 | 43.38 |
|  |  | Relatively high | 323 | 0.55 | 5.88 | 222.03 | 16.67 | 5.15 | 15.32 |
|  |  | High | 35 | 0.15 | 1.57 | 13.49 | 1.01 | 0.34 | 1.01 |
| Environmental carrying capacity | Environmental capacity | Low | 665 | 1.14 | 41.47 | 552.41 | 41.47 | 16.36 | 48.67 |
|  |  | Relatively low | 95 | 0.16 | 4.06 | 54.05 | 4.06 | 1.44 | 4.28 |
|  |  | Medium | 119 | 0.21 | 5.07 | 67.51 | 5.07 | 1.72 | 5.12 |
|  |  | Relatively high | 185 | 0.49 | 6.43 | 85.63 | 6.43 | 2.07 | 6.16 |
|  |  | High | 1314 | 7.34 | 42.98 | 572.54 | 42.98 | 12.02 | 35.77 |
| Ecological carrying capacity | Ecological sensitivity | Low | 278 | 2.24 | 23.99 | 70.22 | 5.27 | 1.22 | 3.62 |
|  |  | Relatively low | 360 | 2.79 | 29.90 | 121.08 | 9.09 | 2.22 | 6.60 |
|  |  | Medium | 868 | 3.02 | 32.32 | 383.24 | 28.77 | 8.49 | 25.25 |
|  |  | Relatively high | 485 | 0.82 | 8.83 | 392.56 | 29.47 | 10.77 | 32.03 |
|  |  | High | 384 | 0.46 | 4.96 | 365.03 | 27.40 | 10.93 | 32.50 |
| Natural disaster risk | Natural disaster risk | Low | 2 | 0.01 | 0.10 | 0.85 | 0.06 | 0.01 | 0.02 |
|  |  | Relatively low | 46 | 0.19 | 2.07 | 21.50 | 1.61 | 0.43 | 1.28 |
|  |  | Medium | 730 | 1.70 | 18.21 | 535.76 | 40.22 | 14.86 | 44.20 |
|  |  | Relatively high | 1330 | 4.82 | 51.56 | 657.00 | 49.32 | 15.63 | 46.50 |
|  |  | High | 267 | 2.62 | 28.05 | 117.02 | 8.78 | 2.69 | 8.01 |
| Sum | | | 2,378 | 9.34 | 100.00 | 1,332.13 | 100.00 | 33.62 | 100.00 |

**Supplemental Table 5**

**Evaluation results of the risk forewarning of regional sustainability based on NCC in China.** The evaluation covers 2,375 districts and counties in China, excluding Hong Kong, Macao, Taiwan, and Xisha, Zhongsha, Dongsha, and Nansha islands, etc. The regional basic geographic data such as administrative divisions are derived from the National Geomatics Center of China. The population data is derived from the Tabulation on the 2010 Population Census of the People’s Republic of China by County. The 2010 GDP data is derived from the National Bureau of Statistics of China. The table is adapted from the REF [9].

| Risk forewarning type | Number of counties and districts | Area | | Population | | GDP | |
| --- | --- | --- | --- | --- | --- | --- | --- |
|  |  | Total  (million km^2^) | Percentage  (%) | Total  (billion people) | Percentage  (%) | Total  (trillion Chinese Yuan) | Percentage  (%) |
| Overload | 197 | 0.26 | 2.84 | 224.30 | 16.84 | 7.34 | 21.84 |
| Critical overload | 431 | 0.79 | 8.42 | 363.47 | 27.29 | 11.63 | 34.60 |
| No overload | 1747 | 8.29 | 88.74 | 744.36 | 55.88 | 14.65 | 43.56 |
| Total | 2375 | 934.22 | 100.00 | 1,332.13 | 100.00 | 33.62 | 100.00 |

**Supplemental Table 6**

**Statistics of each single index overload in the overload and critical overload areas.** The evaluation covers 2,375 districts and counties in China, excluding Hong Kong, Macao, Taiwan, and Xisha, Zhongsha, Dongsha, and Nansha islands, etc. The regional basic geographic data such as administrative divisions are derived from the National Geomatics Center of China. The population data is derived from the Tabulation on the 2010 Population Census of the People’s Republic of China by County. The 2010 GDP data is derived from the National Bureau of Statistics of China. The table is adapted from the REF [9].

| Evaluation | Indices | Number of units | Area | | Population | | GDP | |
| --- | --- | --- | --- | --- | --- | --- | --- | --- |
|  |  |  | Number  (×10^4^ km^2^) | Percent  (%) | Number  (million persons) | Percent  (%) | Number  (billion Chinese yuan) | Percent  (%) |
| Basic evaluation | Development and utilization of water resources | 138 | 19.49 | 73.58 | 123.08 | 54.87 | 3857.19 | 52.53 |
|  | Land resource pressure index | 77 | 12.93 | 48.82 | 126.27 | 56.29 | 4371.32 | 59.54 |
|  | Nonattainment index of pollutant concentration | 129 | 14.09 | 53.19 | 189.06 | 84.29 | 6183.18 | 84.21 |
|  | Ecosystem health | 9 | 5.05 | 19.07 | 2.71 | 1.21 | 38.56 | 0.53 |
| Specific evaluation | Change index of cultivated land quality | 5 | 1.96 | 7.40 | 1.69 | 0.75 | 32.02 | 0.44 |
|  | Grassland livestock balance index | 4 | 1.31 | 4.94 | 1.32 | 0.59 | 26.05 | 0.35 |
|  | Black grey index of water and gas environment | 86 | 7.28 | 27.50 | 70.33 | 31.36 | 2380.19 | 32.42 |
| Sum | | 197 | 26.49 | 100.00 | 224.30 | 100.00 | 7342.39 | 100.00 |

**Reference**

1. Fan J, Wang Y, Wang C *et al.* Reshaping the sustainable geographical pattern: A major function zoning model and its applications in China. *Earth's Future* 2019; **7**: 25-42.

2. Liu B, Siu YL, Mitchell G. A quantitative model for estimating risk from multiple interacting natural hazards: an application to northeast Zhejiang, China. *Stochastic Environmental Research Risk Assessment* 2017; **31**: 1319-1340.

3. Pelling M. *The vulnerability of cities. Natural disasters and social resilience*. Earthscan Publications, 2003.

4. United Nations Intentional Strategy for Disaster Reduction (UNISDR). Living with risk. A global review of disaster reduction initiatives. https://www.undrr.org/publication/living-risk-global-review-disaster-reduction-initiatives (date last accessed).

5. Jensen FV, Nielsen TD. *Bayesian networks and decision graphs*. Springer, 2007.

6. Lauritzen SL. The EM algorithm for graphical association models with missing data. *Computational statistics data analysis* 1995; **19**: 191-201.

7. Fan J. Draft of major function oriented zoning of China. *Acta Geogr Sin* 2015; **70**: 186-201.

8. Research IoGSaNR. Comprehensive Assessment of the Carrying Capacity of Resources and Environment of the Preliminary Research Results of the National Land Planning Outline (2011~2030). 2013.

9. Institute of Geographic Sciences and Natural Resources Research. National scientific assessment report on monitoring and early warning of the carrying capacity of resources and environment. Beijing: Institute of Geographic Sciences and Natural Resources Research, 2014.
